# Scabies and its associated factors among under 15 years children in Wadila district, Northern Ethiopia, 2019

**DOI:** 10.11604/pamj.2020.37.224.25753

**Published:** 2020-11-06

**Authors:** Tefera Haile, Tadesse Sisay, Tadeg Jemere

**Affiliations:** 1 Department of Environmental Health, College of Medicine and Health Sciences, Wollo University, Dessie, Ethiopia,; 2 Department of Biomedical Sciences, College of Health Sciences, Debre Tabor University, Debre Tabor, Ethiopia

**Keywords:** Scabies, children, Ethiopia

## Abstract

**Introduction:**

scabies is a contagious ectoparasite of the skin. It is caused by the mite Sarcoptes scabiei var. hominis that causes a pruritic skin eruption. It was estimated that more than 300 million cases of scabies occur worldwide every year. It remains one of the commonest skin diseases seen in developing countries including Ethiopia. Therefore, the main aim of this study is to determine scabies prevalence and its associated factors among less than 15 years children in Wadila district, Northern Ethiopia.

**Methods:**

community based cross-sectional study was conducted on 583 participants from January 30^th^ to February 28^th^ 2019. Multistage sampling technique was used and data were collected using pre-tested interviewer administered questionnaire. SPSS version 23.0 was used for analysis and bivariable logistic regression was computed and variables having p < 0.25 was modelled in multi-variable logistic regression to control confounders. The level of statistical significance was set at p < 0.05.

**Results:**

the prevalence of scabies infestation was 23.8% in Wadila district. Not using of soap when taking shower [AOR=2.93 (95% CI:1.72-5.00)], using unimproved water source [AOR=1.82 (95% CI:1.04-3.16)], sharing clothes from scabies cases [AOR=10.10 (95% CI: 3.37-30.23)], uncleaning of the house every day [AOR=2.28 (95% CI: 1.32-3.95)], presence of pet animals at home [AOR= 3.01(95% CI: 1.66-5.45)] and went to epidemic areas of scabies [AOR= 4.09 (95% CI: 2.37-7.06) were predictors of scabies infestation.

**Conclusion:**

there was higher prevalence of scabies infestation in Wadila district. Therefore, special attention should be given to under 15 years children.

## Introduction

Scabies is a contagious ectoparasite of the skin. It is caused by the mite *Sarcoptes scabiei var. hominis* that causes a pruritic skin eruption [1]. Nymphs mature in approximately two weeks and then emerge as adults to the surface of the skin, where they mate and (re)invade the skin of the same or another host [2]. Transfer of newly fertilized female mites from person to person occurs mainly by intimate contact and is facilitated by crowding, poor hygiene, and multiple sexual partners [3]. The life cycle of S. *scabiei* is 4-6 weeks and it is an obligate human parasite [2]. The incidence of scabies can increase during natural and manmade disasters. Scabies remains one of the commonest skin diseases seen in developing countries [4]. The severity of scabies infestation is directly related to the number of mites residing on the skin and the length of time between initial infestation and subsequent diagnosis and treatment [5]. It is particularly prevalent in resource-poor conditions and among children, which is associated with insufficient access to health-care subsidies [1, 6].

Globally, scabies is a common cause of itching dermatosis infesting around 300 million persons [7]. It is an important health problem affecting all age groups, both sex and all races [6]. According to the result of different studies, the prevalence of scabies ranges from 2-90.1% [6, 8, 9-13]. Results of different studies revealed that age, residence, education, occupation of caregivers, type of house, number of family size, sleeping with others, family history of itchy rash, sharing clothes with others, and having animals at home were significantly associated with the occurrence of scabies [8, 9, 13]. According to the studies conducted in Ethiopia, there was inconsistency in the finding and little is known about associated factors [13]. Therefore, the main aim of this study is to avoid uncertainty on the findings of previous studies and investigate additional factors associated with scabies infestation.

## Methods

**Study design, area and period:** community based cross-sectional study was conducted in Wadila district which is one of the 11 rural districts of North Wollo Zone, Amhara Regional State. Wadila district is located 245km Northeast of Bahrdar, and 630km from Addis Ababa. The district has 25 (2 urban and 23 rural) Kebeles. The study was conducted from January 30^th^ to February 28^th^ 2019.

**Source and study population:** all under 15 years old population who live in Wadila district were source populations and sample of less than 15 years children in selected kebeles were study populations.

**Eligibility criteria:** all under 15 years children living in the six selected kebeles during study period were included and those who have other skin diseases confirmed by health professional were excluded from the study.

**Sample size determination:** sample size was calculated using single population proportion formula by considering proportion of scabies infestation prevalence 50% (since there is no study done in less than 15 years old children in Ethiopia), margin of error 5%, and confidence level of 95%. Considering the above assumption using the following formula:

$$\text{n=}\frac{(\text{Z}\alpha \text{/2})2\times \text{P(1-P)}}{\text{d2}}$$

n =(1.96x1.96)x0.5x0.5/0.0025=384

Multi-stage sampling technique was used in order to get study participants. Therefore, by using 1.5 as design effect and assuming 5% non-response rate, the final sample size was 605.

**Sampling technique:** multi-stage sampling technique was used so as to select a representative sample of study participants. First from the total 25 kebeles, 6 kebeles were selected randomly. A large proportion of the Ethiopian population (47%) is under age 15 [10]. It was calculated under 15 years children from the total population of each kebeles. Second, sample size was allocated proportionally to each selected kebeles according to population size. Then study participants were selected from 6 kebeles by using systematic sampling method based on household numbers. We computed household interval [K=15] and a lottery method was used to get the first household. Finally, study participants were selected from households by using random sampling method when more than one under 15 years´ children were present in the family. Moreover, during data collection if under 15 years children were not found in the selected households, next household who had under 15 years children was used.

### Variables of the study

**Dependent variable:** scabies infestation.

**Independent variable:** socio-demographic factors (age, sex, number of family size, level of education, income and occupation). Environmental factors, (availability and types of water source, livestock, presence of pet animals, house condition and structure and environmental sanitation). Behavioral factor (sharing of cloth from scabies cases, using soap when taking shower, nutritional status, traveling history, diagnosis time and contact treatment).

### Operational definition

**Scabies infestation:** diagnosis was made based upon presence of the typical rash and symptoms of unrelenting and worsening itch, particularly at night.

**Contact:** a person without signs and symptoms consistent with scabies who has had direct contact (particularly prolonged, direct, skin-to-skin contact) with a suspected or confirmed case in the two months preceding the onset of scabies signs and symptoms in the case [11].

**Infestation:** for persons or animals, the lodgment, development, and reproduction of Arthropods (e.g. lice, fleas, ticks, mites) on the surface of the body or in the clothing [2].

**Improved drinking water sources:** public taps or standpipes, tube wells or boreholes, protected dug wells, protected springs, rainwater collection [12].

**Kebele:** the currently lowest administrative unit within the district in Ethiopian context.

**Nutrition status:** body mass index (BMI) >24.9 kg/m^2^-overweight. BMI=18.5-24.9 kg/m^2^-normal and BMI<18.5 kg/m^2^-underweight. MUAC screen >12cm for children 6–59-month-old normal. MUAC screen <12cm for children 6–59-month-old not normal. MUAC screen >23cm for lactating women having children less than 6 month normal. MUAC screen <23cm for lactating women having children less than 6 month not normal [13].

**Unimproved drinking water sources:** unprotected dug well, unprotected spring, cart with small tank/drum, tanker truck [12].

**Wealth quintile classification:** poorest (lowest) = ≤20%, poor (second) = ≤40%, medium (middle) = ≤60%, rich (fourth) = ≤80%, richest (highest) = >80% [10].

**Data collection procedures:** data were collected using pre-tested interviewer administered questionnaire. Four diploma nurses participated to data collection and two experienced health officers on physical examination. Scabies was diagnosed based on physical examination. If lesions were observed during the physical examination on body surfaces such as interdigital spaces, hand, wrist, arm, elbow, axilla, leg, foot, abdomen, thorax, mamilla/perimammillar area, back, buttock, genital/inguinal area, and head (scalp/neck/face), they were reported as scabies. Disagreements between health officers were reconciled through discussion and consensus. Nutritional status was identified by using digital seca 881 U balance, measurement tape and arm circumference insertion tape based on instruction manual. The household income was measured by a wealth index questioner based on district resources, taken from Ethiopian Demographic Health Survey (EDHS). Data collectors gave Ivermectin for those who had >15 kg weight and having scabies infestation and advise them to visit health institutions for complicated and weight <15 kg cases and lactating women.

**Data analysis procedure:** data were checked for completeness, entered into Epi-data version 3.1 and exported to SPSS version 23 for analyses. Bivariable logistic regression analyses were done to see the association between each independent variable and the outcome variable. Variables with p < 0.25 were entered into multivariable logistic regression to control possible confounders. Odds ratio with its 95% confidence interval was used to identify the determinants of scabies infestation. Level of statistical significance was set at p < 0.05.

**Data quality management:** the questionnaire was translated to Amharic language and then retranslated back to English for its consistency. Pre-test was done on 5% of the total sample size in closest kebeles other than the selected ones for the study. Data collectors and supervisors were trained for two days on the objectives, procedures, and data collection techniques of the study. All under 15 years children from selected households were clinically assessed by using Federal Ministry of Health Multi-Sectorial Scabies Outbreak Emergency Response manual. To minimize bias, respondents were assured to keep their response confidential.

**Ethics approval and consent to participate:** ethical approval was obtained from Ethical Review Committee of Wollo University with ethical approval number of WUPGD/504/19. Written informed consent was taken from the parents of each study participants. Confidentiality of information was kept properly.

## Results

**Socio-demographic characteristics:** a total of 583 participants were included in the study making a response rate of 96.4%. More than half (51.3%) of participants were female. The proportion of children aged less than 5 years was higher when compared with the proportion of children aged 5-9 years and 10-14 years. The mean age of study participants was 6.7 years with a standard deviation of ± 4.2 years. Regarding educational level of the participants´ majority (82.1%) of children attained primary school (age ≥7years) and nearly two-third (61.4%) of the mothers (care givers), were unable to read and write. Farming was the main occupation for the majority (85.6%) of households. The mean family size of households in the district was 5.1 with standard deviation of ± 1.5. In terms of wealth quintile, the study population is poor relative to the general population, but not the poorest of the poor (Table 1).

**Table 1:** socio-demographic characteristics of study participants in Wadila district, Northern Ethiopia, 2019

Variables		Frequency (n)	Percentage (%)
Sex of respondent	Male	284	48.7
	Female	299	51.3
	Total	583	100.0
	Unable to read and write	358	61.4
	Able to read and write	101	17.3
	Primary school	99	17.0
	Secondary school	15	2.6
	More than secondary school	10	1.7
	Total	583	100.0
Education of respondent	Unable to read and write	29	10.6
	Read and write	20	7.3
	Primary school	224	82.1
	Total	273	100.0
Family size category	≤5	345	59.2
	>5	238	40.8
	Total	583	100.0
Age category	Under 5	228	39.1
	5-9	171	29.3
	10- 14	184	31.6
	Total	583	100.0
Primary occupation of household	Farming	499	85.6
	Daily laborer	20	3.4
	Merchant	21	3.6
	Employee	43	7.4
	Total	583	100.0

Note: n=number of study participants

**Environmental characteristics:** nearly two-third (64.8%) of study participants reported the presence of pet animals at home and 60% of them reported that livestock was living within the same household. Regarding housing structure and condition, the study participants reported that, the house structure was constructed from wood with mud for wall (73.4%) and mud for floor and half of corrugated iron for roof (98.1%). Around 29.5% of study participants did not clean their house every day and 84.2% of participants did not keep their environmental sanitation. Regarding to water source, nearly three-fourth (74.1%) of households use improved water source and majority, (84%) households in study kebeles travel less than 30 minutes to fetch water in walking distance from their home.

**Behavioral characteristics:** majority, (62.8%) of participants had poor personal hygiene during observation; whereas, 95% of study participants wash their clothes in their lifetime and 93.7% of them wash their clothes ≤4 times per month. On the other hand, 82% of study participants took shower and only 59.8% of them use soap when taking shower. Nearly 5% of study participants share clothes from scabies cases and 25.4% of study participants had history of travel to epidemic areas of scabies during the last two months. Based on nutritional status, 64.4% of the participants had normal BMI by screen weight for height square for age group ≥5 years children and 91.7% of them had normal for age group < 5 years children by MUAC screen.

**Prevalence of scabies infestation:** the overall prevalence of scabies infestation in Wadila District was 23.84% with the CI of (20.1%-27.1%) and there was a higher prevalence of scabies among male participants than females. From the total 139 scabies cases, 33.7% were found between age groups 10-14 years, 25.5% of cases live in family size category ≤5, and 24.6% of care givers were illiterate (Table 2).

**Table 2:** prevalence of scabies infestation distributed in socio-demographic factors in Wadila district, Northern Ethiopia, 2019

Socio-demographic Variable	Category	Total	Presence of scabies		Percentage (%)
			No	Yes	
**Age**	<5	228	40	188	17.5
	5-9	171	37	134	21.6
	10-14	184	62	122	33.7
**Sex**	Male	284	69	215	24.3
	Female	299	70	229	23.4
**Family size**	≤5	345	88	257	25.5
	>5	238	51	187	21.4
**Education of care giver**	Unable to read and write	358	88	270	24.6
	Able to read and write	101	29	72	28.7
	10 schools	99	19	80	19.2
	20 schools and above	25	3	22	12

**Factors associated with scabies infestation:** all variables that had p-value < 0.25 in bivariable analysis were included in the forward logistic regression model. After adjusting for these variables, not cleaning of the house everyday [AOR: 2.28 CI (1.32-3.95) p=0.003], presence of pet animals at home [AOR= 3.01, CI (1.66-5.45), unimproved water source [AOR=1.82, CI (1.04-3.16), p=0.035], not using of soap while they took shower [AOR: 2.93, CI (1.72-5.00) P= 0.001], went to epidemic area of scabies cases [AOR: 4.09, CI (2.37-7.06),P= 0.001] and sharing of clothes from scabies case [AOR: 10.1, CI (3.4-30.2), P= 0.001] were independent predictors of scabies infestation (Table 3).

**Table 3:** bivariable and multivariable logistic regression model of factors independently associated with scabies infestation among under 15 children at Wadila district, North Ethiopia, 2019

Variables		Scabies infestation		OR (95% CI)	
		Yes N (%)	No N (%)	COR	AOR
Cleaning of the house everyday	Yes	68	343	1	1
	No	71	101	3.55(2.38-5.29)	2.28(1.32-3.95)*
Pet animals at home	Yes	111	267	1	1
	No	28	177	2.63(1.67-4.15)	3(1.66-5.45)*
Water source	Improved	87	345	1	1
	Unimproved	52	99	2.08(1.38-3.14)	1.82(1.04-3.16)*
Use soap when taking shower	Yes	37	249	1	1
	No	68	124	3.69(2.34-5.82)	2.93(1.72-5)*
Went to epidemic area of scabies cases	Yes	76	72	6.23(4.1-9.47)	4.09(2.37-7.06)*
	No	63	372	1	1
Shared clothes from scabies case	Yes	24	8	11.37(4.9-25.9)	10.1(3.4-30.2)*
	No	115	436	1	1

* P-value ≤ 0.05, AOR: Adjusted Odds Ratio, COR: Crude Odds Ratio

## Discussion

Data on prevalence and associated risk factors about scabies infestation in under 15 years children provide valuable information to serve as a basis for methods of prevention, control and therapeutic services. This study showed that, prevalence of scabies infestation was 23.84% in Wadila district. This finding is consistent with study conducted in Gondar town, 22.5% [14]. However, our finding is higher when compared to findings from other countries such as Solomon Islands, 19.2% [15], Fiji, 18.5% [16], Germany 10.5% [6], Nigeria 13.3% [17], Cameroon 17.8% [18] and Dabat 9.3% [19]. But it is lower than from the finding of the studies conducted in India, 90.1% [20] and Pakistan, 38.15% [21], and Illu Aba Bora zone (63.5%) [22]. This discrepancy might be due to the presence of different water, hygiene and sanitation facilities in those countries and might also be related with the difference in sample size, study time, level of awareness and health seeking behavior across these populations, examined population, and the examination method (clinical or microscopic).

The multivariable logistic regression showed that, not cleaning of the house every day leads to scabies infestation. Study participants who were not cleaning their house every day were 2.28 times more likely to develop scabies infestation when compared with those who clean their house every day. This is in line with the study conducted in Pakistan [23]. If the house is not cleaned every day, there will be accumulation of dust particles and this creates favorable environment for reproduction of mites. Unimproved water source was also another predictor of scabies infestation according to this study. Participants who used unimproved water source were 2 times more likely to get scabies infestation when compared with those who used improved water source. According to WHO report, majority (around 80%) of the global population without access to improved drinking-water supplies resides in rural areas [24]. This increases susceptibility to different skin problems like scabies infestation. Safe, adequate and accessible water supply, can help to reduce many of the disease, affecting under-privileged populations [25]. The odds of developing scabies infestation among those who didn´t use soap when taking shower is 2.93 times higher than among those who use soap. This finding is consistent with the study conducted in Nigeria [9]. Probably, using soap is important to remove scabies mites from the body and decrease the risk of transmission.

Scabies infestation was 10 times more likely to develop in children who share clothes from scabies cases when compared to those who didn´t share clothes from scabies cases. This result was consistent with study done in Hadiya Zone, East Badewacho districts [26] and in Pakistan [27]. This is probably, the mites exist on the clothes of scabies cases and this facilitates scabies transmission. Study participants who went to epidemic areas of scabies cases were 4 times more likely to get scabies infestation when compared with who did not went to epidemic areas of scabies cases. This finding is in line with study conducted in Gondar town, Ethiopia [14] and Dabat [19]. This is probably, the healthy persons who went to epidemic areas could share clothes, playing and sleeping with scabies cases and Sarcoptes mites can transmit from scabies cases to healthy person (people) during close contact. The likelihood of children developing scabies infestation in households having pets at home was also 3 times higher than from those living without pets at home. This finding is consistent with study done in Pakistan [20] and India [8]. Probably, presence of pet animal at home can serve as a source and means of reproduction of scabies mites and there might be a zoonotic transmission of scabies mites from these animals to children during contact [5].

**Limitation of the study:** first, diagnosis of scabies was made on the basis of clinical history and physical examination alone. Skin scrapings for direct microscopy and magnification with dermoscopy were not used due to budget constraint. Diagnostic accuracy is dependent upon the clinical perception and experience of the examiner. Second, all environmental factors were not addressed by this study especially moisture content and temperature variation.

## Conclusion

There was higher prevalence of scabies infestation in Wadila district. Not using soap when taking shower, using unimproved water source, sharing clothes from scabies cases, uncleaning of the house every day, went to epidemic areas of scabies cases and presence of pets at home were predictor variables for scabies infestation. Special attention should be given to under 15 years children as they are at the highest risk of infestation.

### What is known about this topic

- Scabies is a common cause of itching dermatosis infesting around 300 million persons worldwide. It is an important health problem affecting all age groups, both sex and all races. According to the result of different studies, the prevalence of scabies ranges up to 90%;
- Scabies prevalence is also well searched in Ethiopian adults; however, it is not well investigated in under 15 years children.

### What this study adds

- This study will add some evidence on the prevalence and associated factors of scabies on under 15 years children who are at higher risk of acquiring this infestation.
